# Women’s Preferences for Home-Based Self-Sampling or Clinic-Based Testing for Cervical Cancer Screening

**DOI:** 10.1001/jamanetworkopen.2025.58841

**Published:** 2026-02-06

**Authors:** Joël Fokom Domgue, Monalisa Chandra, Olajumoke Oladoyin, Manali Desai, Robert Yu, Sanjay Shete

**Affiliations:** 1 Division of Cancer Prevention and Population Sciences, The University of Texas MD Anderson Cancer Center, Houston; 2 Department of Epidemiology, The University of Texas MD Anderson Cancer Center, Houston; 3 Department of Health Promotion and Behavioral Sciences, School of Public Health, The University of Texas Health Science Center at Houston (UTHealth); 4 Department of Biostatistics, The University of Texas MD Anderson Cancer Center, Houston; 5 Department of Epidemiology, Human Genetics and Environmental Sciences, School of Public Health, The University of Texas Health Science Center at Houston (UTHealth)

## Abstract

**Importance:**

While home-based self-sampling for cervical cancer screening is an evidence-based strategy proven to increase screening access and uptake, it is not currently recommended in the US despite recent Food and Drug Administration approval of the first at-home self-sampling device. Little nationally representative research has examined preference for and drivers of home-based self-sampling over clinic-based testing (the standard of care).

**Objective:**

To assess women’s perspectives about, reasons for considering, and factors associated with preferring at-home self-sampling for cervical cancer screening.

**Design, Setting, and Participants:**

This population-based cross-sectional study used data from the 2024 Health Interview National Trends Survey (HINTS 7), a nationally representative survey of the civilian, noninstitutionalized US adult population offered between March and September 2024. Respondents included in this study were individuals aged between 21 and 65 years who were eligible for cervical cancer screening per the US Preventive Services Task Force guidelines and who self-reported their gender identity. Respondents who indicated not needing cervical cancer screening or who did not report their preference for any screening modality (home-based self-sampling or clinician-collected sampling) were excluded. Data were analyzed from May 12 to 25, 2025.

**Exposures:**

Age, race and ethnicity, income, educational level, sexual orientation, marital status, health insurance, urbanicity of residence, trust in the health care system, past-year number of visits to a health care practitioner, and prior experience of discrimination or prejudice when getting medical care.

**Main Outcomes and Measures:**

The main outcome was preference for at-home vaginal self-sampling over clinic-based testing, measured using the HINTS 7 question, “If you had choice, how would you prefer to do the cervical cancer screening test?” Responses were: preference to have a health care practitioner do the test in his or her office, preference to self-collect specimen for the test at home, not knowing which option to choose, and not applicable. Weights were assigned to improve representativeness of the general US adult population. The proportion of individuals who reported preferring either screening modality was estimated using weighted percentages. Survey-weighted odds ratios (ORs), adjusted for covariates, were calculated to identify factors associated with preference for at-home self-sampling.

**Results:**

Among the 2300 women included (mean [SD] age, 45.5 [29.2] years), most were married or living as married (weighted percentage, 58.2% [95% CI, 56.5%-60.0%]), health insured (91.9%; 95% CI, 90.7%-93.1%), and educated up to some college (61.6%; 95% CI, 60.1%-63.0%). Overall, 462 (20.4%; 95% CI, 17.4%-23.4%) preferred at-home self-sampling, 1402 (60.8%; 95% CI, 57.2%-64.4%) preferred clinic-based testing, and 436 (18.8%; 95% CI, 15.5%-22.1%) were uncertain about their choice. Non-Hispanic Black respondents (adjusted OR [AOR], 0.45; 95% CI, 0.21-0.96) had lower odds of preferring at-home self-sampling compared with non-Hispanic White individuals. Women who had experienced prejudice or discrimination when getting medical care had higher odds (AOR, 1.94; 95% CI, 1.16-3.22) of preferring at-home self-sampling. The most commonly self-reported reasons for preferring at-home self-sampling were privacy (54.9%; 95% CI, 49.8%-60.0%), time constraints (35.1%; 95% CI, 29.0%-41.2%), and fear of embarrassment (33.4%; 95% CI, 28.0%-38.8%).

**Conclusions and Relevance:**

In this cross-sectional study, marginalized populations, individuals with low income, and individuals who do not trust the health care system were more likely to prefer at-home self-sampling for cervical cancer screening or not know which option to choose. To address cervical cancer inequities and increase screening uptake, the findings suggest US guidelines should incorporate home-based self-sampling as an alternative to clinic-based testing, women’s education and empowerment should be enhanced, and tailored interventions focusing on high-risk groups are needed to increase awareness and self-confidence in performing home-based self-sampling.

## Key Points

**Question:**

Do women prefer home-based self-sampling over clinic-based testing for cervical cancer screening, and what factors are associated with this preference?

**Findings:**

In this population-based cross-sectional study of 2300 screening-eligible women, 20% preferred home-based self-sampling, 61% preferred clinic-based testing, and 19% were uncertain. Non-Hispanic Black compared with non-Hispanic White individuals had lower odds of preferring home-based self-sampling, while individuals who had experienced prejudice or discrimination in medical settings had higher odds.

**Meaning:**

Fewer individuals preferred home-based vs clinic-based testing, but higher odds of preferring home-based self-sampling among marginalized groups suggest that if incorporated into national guidelines, the home-based modality could increase cervical cancer screening uptake.

## Teaser

This cross-sectional study assesses whether women in the US prefer at-home self-sampling or clinic-based testing for cervical cancer screening and factors associated with this preference.

## Introduction

Over the past half-century, the implementation of cytology-based organized screening programs in high-income settings has led to significant declines in the burden of cervical cancer.^1^ After 2 decades of upward trends, screening coverage in the US decreased from 86.5% in 2000 to 75.8% in 2023.^2^ Resultantly, the observed reduction in cervical cancer incidence has gradually reversed, especially in women aged 30 to 44 years, with yearly rates increasing by 1.7% between 2012 and 2019 in this age range.^3^ According to the US National Cancer Institute, nearly 13 500 new cases and 4500 deaths from cervical cancer were expected in 2025.^4^ This is a matter of concern, because most cervical cancer cases and deaths in the US occur in women from socioeconomically disadvantaged and minority groups^5^ who are not up-to-date with screening guidelines.^6,7^

The development of human papillomavirus (HPV) tests, which have consistently been shown to be more sensitive and more specific than cytology in detecting precancerous cervical lesions,^8,9^ prompted the US Preventive Services Task Force (USPSTF) in 2018 and the American Cancer Society (ACS) in 2020 to endorse primary screening with HPV testing of clinician-collected samples as a recommended or preferred cervical cancer screening strategy in the US.^10,11^ A key advantage of HPV testing over cytology is that HPV testing can be performed on specimens collected by women themselves (as opposed to samples collected by health care practitioners) without losing its diagnostic accuracy.^12,13^ By providing more privacy and convenience to individuals, at-home self-sampling offers an alternative to conventional clinic-based testing for primary screening, as it has the potential to address many of the known barriers to practitioner-collected sampling, including embarrassment, lack of trust in the health care system, logistic issues such as difficulty in scheduling or attending physician visits, time constraints, transportation challenges, and remoteness from health care facilities.^6,14,15,16^ Although previous clinical trials have concluded that a screening approach grounded in mailing home-based self-collection kits is feasible in the US and could improve screening participation among underscreened women,^17,18,19^ self-sampling is yet to be endorsed by the USPSTF guidelines.

On May 9, 2025, the Food and Drug Administration (FDA) approved the first at-home self-sampling device for cervical cancer screening in the US.^20^ This regulatory authorization is a major milestone toward the introduction of home-based tests into screening guidelines to further improve cervical cancer screening access and uptake in high-risk populations. Considering the clinical validity and cost-effectiveness of at-home self-sampling, its adoption by cervical cancer screening programs requires a better understanding of women’s perspectives about this innovative strategy. So far, only a few US-based studies have assessed the acceptability of or preferences for at-home self-sampling in specific groups of women screened for cervical cancer.^19,21,22,23,24^ These studies have established greater preference among low-income and underscreened women for home-based self-sampling,^23,24^ reported high tolerance (no or infrequent and minor adverse effects) among women for HPV self-sampling,^19,22,24^ and identified increased convenience and reduced embarrassment as common benefits of HPV self-sampling.^21,24^ However, they often were limited to a subset of women who had been invited to perform self-sampling due to nonadherence to screening guidelines, evaluated women’s perceptions of and experience with self-sampling in controlled settings, or did not include women who had not consented to use HPV self-sampling devices. To our knowledge, no previous research has examined women’s preferences for home-based self-sampling over clinic-based testing using a nationally representative sample of US women while accounting for the perceived benefits of this alternative screening modality. To further inform the future incorporation of HPV self-sampling into US cervical cancer screening guidelines, we carried out this population-based study to (1) assess women’s perspectives about at-home self-sampling, (2) identify self-reported reasons for considering this innovative sampling approach, and (3) examine factors associated with preferring at-home self-sampling over clinician-collected sampling for cervical cancer screening in the US.

## Methods

### Data Source and Study Design

This cross-sectional study was based on data from the 2024 Health Information National Trends Survey (HINTS 7), a nationally representative survey of US adults in the civilian noninstitutionalized population.^25^ The survey was offered in English and Spanish between March and September 2024 using a dual-mode method: self-administered paper questionnaires mailed to residential addresses and an optional web-based response system. Data were released on May 9, 2025. Since HINTS data are deidentified and publicly available, The University of Texas MD Anderson Cancer Center exempted this study from institutional review board review or informed consent.^26^ A total of 7278 individuals aged 18 years or older responded to the HINTS 7 (27.3% response rate).^25^ We included all women aged 21 to 65 years who completed the survey if they were eligible for cervical cancer screening per the USPSTF guidelines and if they responded to the question regarding their perspectives about cervical cancer screening modalities. Our study adhered to the Strengthening the Reporting of Observational Studies in Epidemiology (STROBE) reporting guidelines.^27^

### Conceptual Framework for Measures

The socioecological model informed the assessment of cervical cancer screening preferences in our study and guided the selection of variables.^28^ This model offers a conceptual framework that integrates multiple factors influencing cervical cancer screening at the individual (sociodemographic characteristics), interpersonal (perceived discrimination), organizational (number of follow-up visits needed), community (place of residence), and policy (trust in the health care system) levels to understand why women get screened for cervical cancer.^28^ A key consideration of the socioecological model is that the adoption of health behaviors arises from multifaceted interactions across multiple levels, underscoring that broader cultural and socioeconomic environments shape individual health decisions.

### Outcome Variable and Study Population

Our primary outcome was preference for at-home self-sampling over clinician-collected sampling, which was measured using the following question: “If you had choice, how would you prefer to do the cervical cancer screening test?” The possible responses were “Not applicable—I do not need cervical cancer screening,” “I would prefer to have a health professional do the test in a doctor’s office (as happens now)” [hereafter, *clinic-based testing*], “I would prefer to do the test myself at home” [hereafter, *home-based self-sampling*], and “I don’t know which option I would choose.” Among the HINTS 7 participants who were aged 21 to 65 years, all male participants, participants who self-reported their gender identity as “don’t know” or “prefer not to respond,” participants with missing data about their gender, and female participants who did not respond to the question about their preference for any cervical cancer screening modality were excluded from our study. Female respondents who selected “Not applicable—I do not need cervical cancer screening” as the response to this question were also excluded from the study sample and from our analyses, as they likely represented individuals who did not have a cervix at the time of survey (women with a history of hysterectomy or transgender women). According to recent estimates, 0.8% of the US adult population identify as transgender people (one-third of whom consider themselves transgender women),^29^ and 14.6% of US adult women have received a hysterectomy.^30^

### Covariates

Selected sociodemographic variables, including self-reported age (21-29 years, 30-49 years, or 50-65 years), race and ethnicity, marital status (married or living as married; single, separated, divorced, or widowed), household annual income (<$50 000, $50 000 to <$75 000, or ≥$75 000), educational level (up to high school, post–high school or some college; college graduate; or postgraduate education), health care insurance (yes, no), sexual orientation (straight [heterosexual]; gay, lesbian, or bisexual; or used a different term or did not know), and urbanicity of place of residence (rural, urban) were considered as covariates in this study. For race and ethnicity (included in the analysis because cervical cancer screening uptake has been consistently reported to vary according to race and ethnicity in the US^31,32,33,34,35^), the Hispanic category included all individuals who selected “Hispanic” as their ethnicity, regardless of their race; the other categories were non-Hispanic Asian [hereafter, Asian], non-Hispanic Black [hereafter, Black], non-Hispanic White [hereafter, White], and other non-Hispanic race (included individuals of the following minority race groups who reported their ethnicity as non-Hispanic: American Indian or Alaska Native; Guamanian or Chamorro; or Native Hawaiian, Samoan, or Other Pacific Islander). Other factors that could affect respondents’ perception of or connection with health care facilities and/or practitioners were also included: trust in the health care system, including hospitals, pharmacies, and other organizations involved in health care (no trust at all or a little; some trust or a lot); number of visits to a health care practitioner in the past 12 months to receive medical care, not counting emergency room visits (0, 1, or ≥2); and experience of prejudice or discrimination when getting medical care (yes, no). All covariates were selected a priori from the HINTS 7 dataset, based on the socioecological model and prior associations with cervical cancer screening.^31,32,33,34,35^

### Assessment of Self-Reported Reasons for Considering At-Home Self-Sampling

In women who preferred at-home self-sampling or did not know which option to choose, the reasons for considering at-home self-sampling were assessed using the following question: “What are the reasons you would consider collecting your own at-home sample for cervical cancer screening?” Possible responses to this question were “Prefer not to take time off work,” “Save transportation costs,” “I live far from my health care provider,” “privacy,” “to avoid embarrassment,” and “other reasons not listed.” Participants who responded to this question could select more than 1 reason.

### Weights Definition and Variance Estimation

Sampling weights were assigned to each sampled individual, and analyses were performed using these weights to improve the sample’s representativeness of the general US adult population, account for unequal selection probabilities (eg, oversampling racial and ethnic groups and rural participants), adjust for nonresponse, and reduce sampling error. According to the HINTS 7 methodology report, every respondent was assigned a full-sample weight, the computation of which included (1) assigning a household-level base weight, (2) adjusting for household nonresponse, (3) assigning a person-level initial weight, and (4) calibrating person-level weights to population counts.^25^ Furthermore, every respondent was given a set of 50 replicate weights. Replicate weights were calculated using the delete-1 jackknife replicate approach, which yielded robust variance and SE estimates,^36^ accounting for the complex survey design of the HINTS 7. The full sample weights were used to calculate population estimates, and replicate weights were used to compute SEs of these estimates.

### Statistical Analysis

The study population was classified into 3 categories according to women’s perspectives about cervical cancer screening modalities: those who preferred at-home self-sampling (home-based self-collection), those who preferred in-office clinician-collected sampling (physician’s office collection), and those who did not know which one to choose (uncertain). Unweighted and survey-weighted descriptive statistics were used to characterize the study population according to their preference for either of the 2 cervical cancer screening modalities considered (at-home self-sampling and clinic-based testing). A weighted multinomial logistic regression analysis using survey procedures in SAS, version 9.4 (SAS Institute Inc), was performed to examine factors associated with preferring at-home self-sampling for cervical cancer screening, with the aforementioned covariates included in the model. The outcome variable in the multinomial regression was the 3-category variable representing preferences for sample collection, with the reference category being preference for clinician-collected sampling in a physician’s office (the current standard of care) and the comparison categories being preference for at-home self-sampling and not knowing which option to choose. In the survey-weighted multinomial logistic regression analysis, we incorporated the previously described complex survey sampling design of the HINTS 7^25^ to examine factors associated with preference for at-home self-sampling in the study population. Adjusted odds ratios (AORs) with 95% CIs were computed. Statistical tests were 2-tailed, and *P* < .05 was considered statistically significant. Data were analyzed from May 12 to 25, 2025.

## Results

### Characteristics of the Study Population

Among the 7278 participants in the HINTS 7, 4224 (58.0%) were aged 21 to 65 years. Of those, we excluded all male participants (n = 1583 [37.5%]), participants who self-reported their gender identity as “don’t know” or “prefer not to respond” (n = 29 [0.7%]), and participants with missing data about their gender (n = 10 [0.2%]). The remaining 2602 respondents (61.6%) were women who were potentially eligible for cervical cancer screening. We excluded those who did not respond to the question about their preference for any cervical cancer screening modality (n = 25 [1.0%]) or who selected “not applicable—I do not need cervical cancer screening” to this question (n = 277 [10.6%]), resulting in a total of 2300 screening-eligible respondents included in this study (mean [SD] age, 45.5 [29.2] years). The weighted percentage of Asian individuals was 4.3% (95% CI, 3.0%-5.5%); Black individuals, 11.1% (95% CI, 9.9%-12.2%); White individuals, 62.2% (95% CI, 60.3%-64.1%); and individuals of other non-Hispanic race, 4.4% (95% CI, 3.1%-5.7%). A total of 87.2% (95% CI, 85.2%-89.2%) identified as heterosexual and 7.8% (95% CI, 6.1%-9.5%) as lesbian, gay, or bisexual. Most individuals were married or living as married (58.2%; 95% CI, 56.5%-60.0%), had a yearly household income of $50 000 to less than $75 000 (14.5%; 95% CI, 12.1%-16.8%) or $75 000 or more (48.2%; 95% CI, 45.5%-50.9%), had an educational level of up to some college (61.6%; 95% CI, 60.1%-63.0%), were urban dwellers (85.6%; 95% CI, 83.2%-88.1%), and had health insurance (91.9%; 95% CI, 90.7%-93.1%) (Table 1). In addition, a majority of respondents (78.1%; 95% CI, 75.5%-80.8%) reported at least 2 visits to health care practitioners in the past 12 months to receive care outside emergency settings, 77.1% (95% CI, 74.1%-80.0%) reported a lot of or some trust in the health care system, and 76.1% (95% CI, 73.0%-79.2%) reported having never experienced prejudice or discrimination when getting medical care (Table 1).

**Table 1.** Characteristics of the Study Population Overall and by Preference for Clinic-Based Testing or Home-Based Self-Sampling for Cervical Cancer Screening in the 2024 HINTS

Characteristic	Participants							
	Total		Preference for clinic-based testing		Preference for home-based self-sampling		Not sure which option to choose	
	No. (unweighted %)	Weighted % (95% CI)	No. (unweighted %)	Weighted % (95% CI)	No. (unweighted %)	Weighted % (95% CI)	No. (unweighted %)	Weighted % (95% CI)
All participants	2300 (100)	NA	1402 (60.9)	60.8 (57.2-64.4)	462 (20.1)	20.4 (17.4-23.4)	436 (20.0)	18.8 (15.5-22.1)
Age, y								
21-29	311 (13.5)	17.2 (14.5-19.8)	193 (62.1)	58.2 (46.2-70.3)	55 (17.7)	19.2 (7.3-31.1)	63 (20.3)	22.6 (11.5-33.6)
30-49	1035 (45.0)	47.0 (44.1-49.8)	626 (60.5)	62.8 (57.4-68.2)	213 (20.6)	19.6 (15.3-23.8)	196 (18.9)	17.6 (13.7-21.6)
50-65	954 (41.5)	35.9 (33.1-38.7)	583 (61.1)	59.4 (54.8-64.0)	194 (20.3)	22.1 (18.4-25.8)	177 (18.6)	18.5 (14.5-22.6)
Race and ethnicity								
Hispanic	554 (24.6)	18.1 (16.3-20.0)	340 (61.4)	65.5 (59.1-72.0)	93 (16.8)	15.7 (10.2-21.2)	121 (21.8)	18.8 (13.7-23.9)
Non-Hispanic								
Asian	115 (5.1)	4.3 (3.0-5.5)	73 (63.5)	66.1 (51.1-81.1)	17 (14.8)	13.8 (3.9-23.7)	25 (21.7)	20.0 (7.7-32.4)
Black	377 (16.7)	11.1 (9.9-12.2)	268 (71.1)	67.2 (59.0-75.4)	45 (11.9)	15.5 (7.4-23.6)	64 (17.0)	17.3 (12.1-22.5)
White	1114 (49.4)	62.2 (60.3-64.1)	636 (57.1)	57.7 (52.5-62.9)	276 (24.8)	23.6 (19.3-27.9)	202 (18.1)	18.7 (14.2-23.3)
Other^a^	94 (4.2)	4.4 (3.1-5.7)	55 (58.5)	70.3 (56.3-84.3)	24 (25.5)	19.6 (7.8-31.3)	15 (16.0)	10.2 (2.9-17.4)
Sexual orientation								
Lesbian, gay, or bisexual	194 (8.6)	7.8 (6.1-9.5)	95 (49.0)	49.9 (37.0-62.9)	58 (29.9)	30.2 (19.1-41.3)	41 (21.1)	19.9 (7.5-32.2)
Straight (heterosexual)	1966 (86.7)	87.2 (85.2-89.2)	1238 (63.0)	63.2 (59.5-66.9)	375 (19.1)	19.6 (16.5-22.8)	353 (18.0)	17.2 (14.1-20.2)
Used a different term or did not know	107 (4.7)	5.0 (3.5-6.6)	47 (43.9)	32.4 (17.5-47.2)	25 (23.4)	22.3 (6.6-38.0)	35 (32.7)	45.3 (23.4-67.2)
Household annual income								
<$50 000	843 (37.7)	37.3 (34.6-40.0)	494 (36.1)	54.2 (46.5-61.8)	153 (33.9)	20.0 (12.5-27.5)	196 (47.0)	25.8 (17.8-33.9)
$50 000 to <$75 000	376 (16.8)	14.5 (12.1-16.8)	231 (16.9)	59.7 (50.5-68.9)	79 (17.5)	25.1 (16.2-34.0)	66 (15.8)	15.2 (8.8-21.5)
≥$75 000	1016 (45.5)	48.2 (45.5-50.9)	642 (47.0)	66.7 (61.9-71.5)	219 (48.6)	19.4 (15.5-23.3)	155 (37.2)	13.9 (10.8-17.0)
Educational level								
Up to high school, post–high school, or some college	1029 (44.9)	61.6 (60.1-63.0)	597 (58.0)	58.2 (52.7-63.6)	197 (19.1)	20.6 (15.9-25.2)	235 (22.8)	21.3 (16.0-26.5)
College graduate	764 (33.4)	22.2 (20.4-24.1)	494 (64.7)	63.3 (58.5-68.1)	149 (19.5)	20.9 (17.2-24.7)	121 (15.8)	15.8 (11.6-19.9)
Postgraduate education	497 (21.7)	16.2 (14.5-17.9)	303 (61.0)	67.0 (61.5-72.5)	116 (23.3)	19.4 (15.1-23.7)	78 (15.7)	13.6 (8.8-18.4)
Marital status								
Married or living as married	1188 (51.8)	58.2 (56.5-60.0)	739 (62.2)	62.7 (58.4-66.9)	238 (20.0)	20.6 (17.5-23.7)	211 (17.8)	16.7 (13.3-20.2)
Divorced, widowed, separated, or single	1107 (48.2)	41.8 (40.0-43.5)	659 (59.5)	58.1 (51.7-64.4)	224 (20.2)	20.2 (14.1-26.3)	224 (20.2)	21.7 (15.8-27.6)
Urbanicity of place of residence								
Urban	1995 (86.7)	85.6 (83.2-88.1)	1223 (61.3)	61.8 (57.8-65.8)	399 (20.0)	19.2 (16.3-22.2)	373 (18.7)	19.0 (15.2-22.7)
Rural	305 (13.3)	14.4 (11.9-16.8)	179 (58.7)	54.9 (46.1-63.6)	63 (20.7)	27.2 (18.4-36.0)	63 (20.7)	17.9 (11.7-24.0)
Health insurance								
Yes	2026 (88.4)	91.9 (90.7-93.1)	1260 (62.2)	61.7 (57.8-65.6)	406 (20.0)	20.3 (17.3-23.4)	360 (17.8)	18.0 (14.7-21.2)
No	266 (11.6)	8.1 (6.9-9.3)	137 (51.5)	51.1 (39.1-63.1)	55 (20.7)	21.0 (12.3-29.7)	74 (27.8)	27.9 (18.5-37.3)
Visits to a health care practitioner in the past 12 mo, No.								
0	237 (10.3)	10.1 (7.7-12.5)	117 (49.4)	41.6 (29.6-53.6)	50 (21.1)	20.7 (11.7-29.7)	70 (29.5)	37.7 (22.5-52.9)
1	280 (12.2)	11.7 (9.7-13.7)	151 (53.9)	50.3 (39.5-61.0)	67 (23.9)	24.5 (16.5-32.5)	62 (22.1)	25.3 (16.3-34.3)
≥2	1780 (77.5)	78.1 (75.5-80.8)	1132 (63.6)	64.9 (61.1-68.6)	345 (19.4)	19.8 (16.4-23.2)	303 (17.0)	15.3 (12.1-18.6)
How much do you trust the health care system?								
A lot or some	1840 (80.2)	77.1 (74.1-80.0)	1187 (64.5)	64.3 (60.5-68.2)	335 (18.2)	19.4 (16.7-22.1)	318 (17.3)	16.3 (13.1-19.4)
A little or not at all	454 (19.8)	22.9 (20.0-25.9)	212 (46.7)	49.3 (41.2-57.3)	125 (27.5)	23.3 (16.2-30.5)	117 (25.8)	27.4 (18.8-36.0)
Have you ever experienced prejudice or been discriminated against when getting medical care?								
Yes	578 (25.2)	23.9 (20.8-27.0)	308 (53.3)	47.5 (39.9-55.0)	165 (28.6)	28.6 (21.5-35.7)	105 (18.2)	23.9 (14.9-32.9)
No	1712 (74.8)	76.1 (73.0-79.2)	1091 (63.7)	65.1 (60.8-69.4)	295 (17.2)	17.8 (14.8-20.9)	326 (19.0)	17.1 (13.7-20.4)

Abbreviations: HINTS, Health Interview National Trends Survey; NA, not applicable.

^a^ Included individuals of the following minority race groups who reported their ethnicity as non-Hispanic: American Indian or Alaska Native; Guamanian or Chamorro; or Native Hawaiian, Samoan, or Other Pacific Islander.

### Preference for At-Home Self-Sampling Over In-Office Clinician-Collected Sampling

Overall, 462 women (weighted proportion, 20.4%; 95% CI, 17.4%-23.4%) preferred at-home self-sampling for cervical cancer screening, 1402 (60.8%; 95% CI, 57.2%-64.4%) preferred clinician-collected sampling in a physician’s office, and 436 (18.8%; 95% CI, 15.5%-22.1%) were uncertain about their choice (Table 1). The proportion of women who preferred at-home self-sampling was higher among White respondents (23.6%; 95% CI, 19.3%-27.9%) compared with Asian (13.8%; 95% CI, 3.9%-23.7%), Black (15.5%; 95% CI, 7.4%-23.6%), and Hispanic (15.7%; 95% CI, 10.2%-21.2%) respondents and those of other non-Hispanic race (19.6%; 95% CI, 7.8%-31.3%) and was higher among rural dwellers (27.2%; 95% CI, 18.4%-36.0%) compared with urban dwellers (19.2%; 95% CI, 16.3%-22.2%). The proportion of women who preferred at-home self-sampling was lower among those who reported no visit to a health care practitioner in the past 12 months (20.7%; 95% CI, 11.7%-29.7%) compared with those who had 1 physician’s visit (24.5%; 95% CI, 16.5%-32.5%); among individuals who had a lot of or some trust in the health care system (19.4%; 95% CI, 16.7%-22.1%) compared with those who had a little trust or none at all (23.3%; 16.2%-30.5%); and among those who had never experienced prejudice or discrimination when seeking medical care (17.8%; 95% CI, 14.8%-20.9%) compared with those who had ever been discriminated against (28.6%; 95% CI, 21.5%-35.7%) (Table 1).

### Factors Associated With Preferring At-Home Self-Sampling for Cervical Cancer Screening

In the multinomial logistic regression, we compared women who preferred in-office clinician-collected sampling with those who preferred at-home self-sampling and those who did not know which option to choose. In-office clinician-collected sampling (standard of care) was the reference category (Table 2).

**Table 2.** Factors Associated With Preference for Home-Based Self-Sampling for Cervical Cancer Screening Among Eligible Women in the 2024 HINTS

Factor	If you had choice, how would you prefer to do the cervical cancer screening test?^a^			
	Preference for home-based self-sampling		Not sure which option to choose	
	AOR (95% CI)^b^	*P* value	AOR (95% CI)^b^	*P* value
Age, y				
21-29	0.72 (0.26-1.96)	.51	0.68 (0.41-1.11)	.12
30-49	0.78 (0.51-1.21)	.27	0.74 (0.47-1.15)	.17
50-65	1 [Reference]	NA	1 [Reference]	NA
Race and ethnicity				
Hispanic	0.53 (0.28-1.02)	.06	0.59 (0.36-0.98)	.04
Non-Hispanic				
Asian	0.51 (0.18-1.46)	.20	0.78 (0.28-2.19)	.63
Black	0.45 (0.21-0.96)	.04	0.56 (0.30-1.02)	.06
White	1 [Reference]	NA	1 [Reference]	NA
Other^c^	0.69 (0.26-1.86)	.46	0.39 (0.15-0.99)	.047
Educational level				
Up to high school, post–high school, or some college	0.93 (0.56-1.55)	.78	0.96 (0.56-1.67)	.89
College graduate	1 [Reference]	NA	1 [Reference]	NA
Postgraduate education	0.81 (0.54-1.20)	.29	0.83 (0.44-1.59)	.57
Household annual income				
<$50 000	1.20 (0.59-2.47)	.61	1.93 (1.04-3.58)	.04
$50 000 to <$75 000	1.21 (0.61-2.39)	.58	1.03 (0.52-2.06)	.93
≥$75 000	1 [Reference]	NA	1 [Reference]	NA
Sexual orientation				
Straight (heterosexual)	1 [Reference]	NA	1 [Reference]	NA
Lesbian, gay, or bisexual	1.74 (0.85-3.56)	.13	1.29 (0.57-2.93)	.54
Used a different term or did not know	2.16 (0.73-6.34)	.16	3.14 (1.36-7.23)	.008
Urbanicity of place of residence				
Urban	1 [Reference]	NA	1 [Reference]	NA
Rural	1.48 (0.84-2.61)	.17	1.01 (0.55-1.83)	.99
Health insurance				
Yes	1 [Reference]	NA	1 [Reference]	NA
No	1.30 (0.63-2.68)	.47	1.36 (0.75-2.47)	.30
Marital status				
Married or living as married	1 [Reference]	NA	1 [Reference]	NA
Divorced, widowed, separated, or single	0.97 (0.66-1.44)	.89	0.98 (0.63-1.54)	.93
Visits to a health care practitioner in the past 12 mo, No.				
0	1 [Reference]	NA	1 [Reference]	NA
1	1.08 (0.53-2.19)	.83	0.81 (0.35-1.86)	.61
≥2	0.59 (0.30-1.18)	.13	0.37 (0.18-0.75)	.007
How much do you trust the health care system?				
A lot or some	1 [Reference]	NA	1 [Reference]	NA
A little or not at all	1.39 (0.83-2.33)	.20	1.80 (1.12-2.88)	.02
Have you ever experienced prejudice or been discriminated against when getting medical care?				
Yes	1.94 (1.16-3.22)	.01	1.46 (0.80-2.69)	.21
No	1 [Reference]	NA	1 [Reference]	NA

Abbreviations: AOR, adjusted odds ratio, NA, not applicable; HINTS, Health Interview National Trends Survey.

^a^ Reference category was preference for clinic-based testing.

^b^ AORs were adjusted for all covariates.

^c^ Included individuals of the following minority race groups who reported their ethnicity as non-Hispanic: American Indian or Alaska Native; Guamanian or Chamorro; or Native Hawaiian, Samoan, or Other Pacific Islander.

#### In-Office Clinician-Collected Sampling vs At-Home Self-Sampling

Compared with White respondents, Black respondents had lower odds of preferring at-home self-sampling over practitioner collection in a physician’s office (AOR, 0.45; 95% CI, 0.21-0.96; *P* = .04). Women who reported having ever experienced prejudice or discrimination when getting medical care had higher odds (AOR, 1.94; 95% CI, 1.16-3.22; *P* = .01) of preferring at-home self-sampling over practitioner collection in a physician’s office compared with women who had never experienced such prejudice or discrimination. Age, income, educational level, marital status, urbanicity, insurance coverage, and sexual orientation were not associated with preferring at-home self-sampling over clinician collection in a physician’s office (Table 2).

#### In-Office Clinician-Collected Sampling vs Uncertainty About Which Option to Choose

Compared with White respondents, Hispanic respondents (AOR, 0.59; 95% CI, 0.36-0.98; *P* = .04) and those of other non-Hispanic race (AOR, 0.39; 95% CI, 0.15-0.99; *P* = .047) had lower odds of being uncertain about their choice of preferring at-home self-sampling or practitioner collection in a physician’s office. Compared with heterosexual women, those who reported a different term or did not know their sexual orientation had higher odds (AOR, 3.14; 95% CI, 1.36-7.23; *P* = .008) of being uncertain about their choice. Women with a yearly household income of less than $50 000 had higher odds (AOR, 1.93; 95% CI, 1.04-3.58; *P* = .04) of being uncertain about their choice compared with those with a household income of $75 000 or more. Compared with women who reported no visit to a health care practitioner in the past 12 months, those who visited a health care practitioner 2 times or more had lower odds (AOR, 0.37; 95% CI, 0.18-0.75; *P* = .007) of being uncertain about their choice. Women who reported little trust or none at all in the health care system had higher odds (AOR, 1.80; 95% CI, 1.12-2.88; *P* = .02) of being uncertain about their choice compared with those who reported a lot of or some trust in the health care system. Age, educational level, marital status, urbanicity, and insurance status were not associated with being uncertain about preferring at-home self-sampling or practitioner collection in a physician’s office (Table 2).

### Self-Reported Reasons for Considering At-Home Self-Sampling for Cervical Cancer Screening

**Figure 1.**
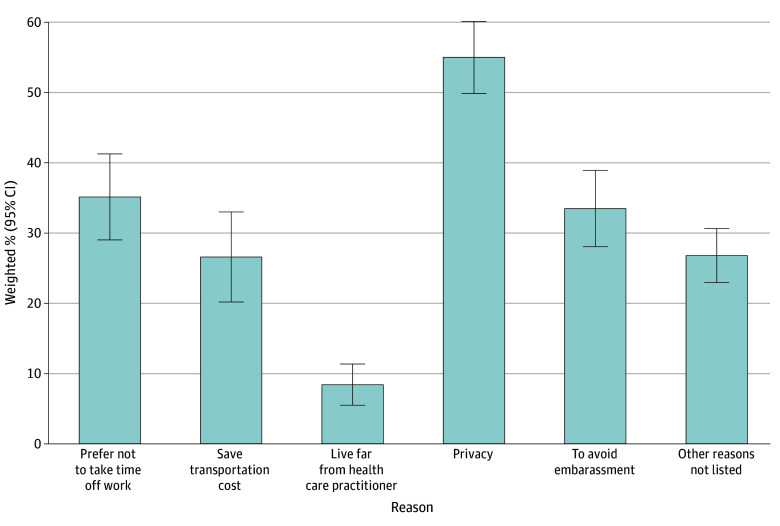
Bar Graph of Reasons for Considering Home-Based Self-Sampling for Cervical Cancer Screening Among Eligible US Women in the 2024 Health Interview National Trends Survey Error bars represent 95% CIs. Numerators were the number of participants who selected each reason for considering at-home self-sampling and denominators were the total number of participants who responded to the question and preferred at-home self-sampling or did not know which option to choose. Because respondents could select more than 1 response, percentages sum to more than 100.

**Figure 2.**
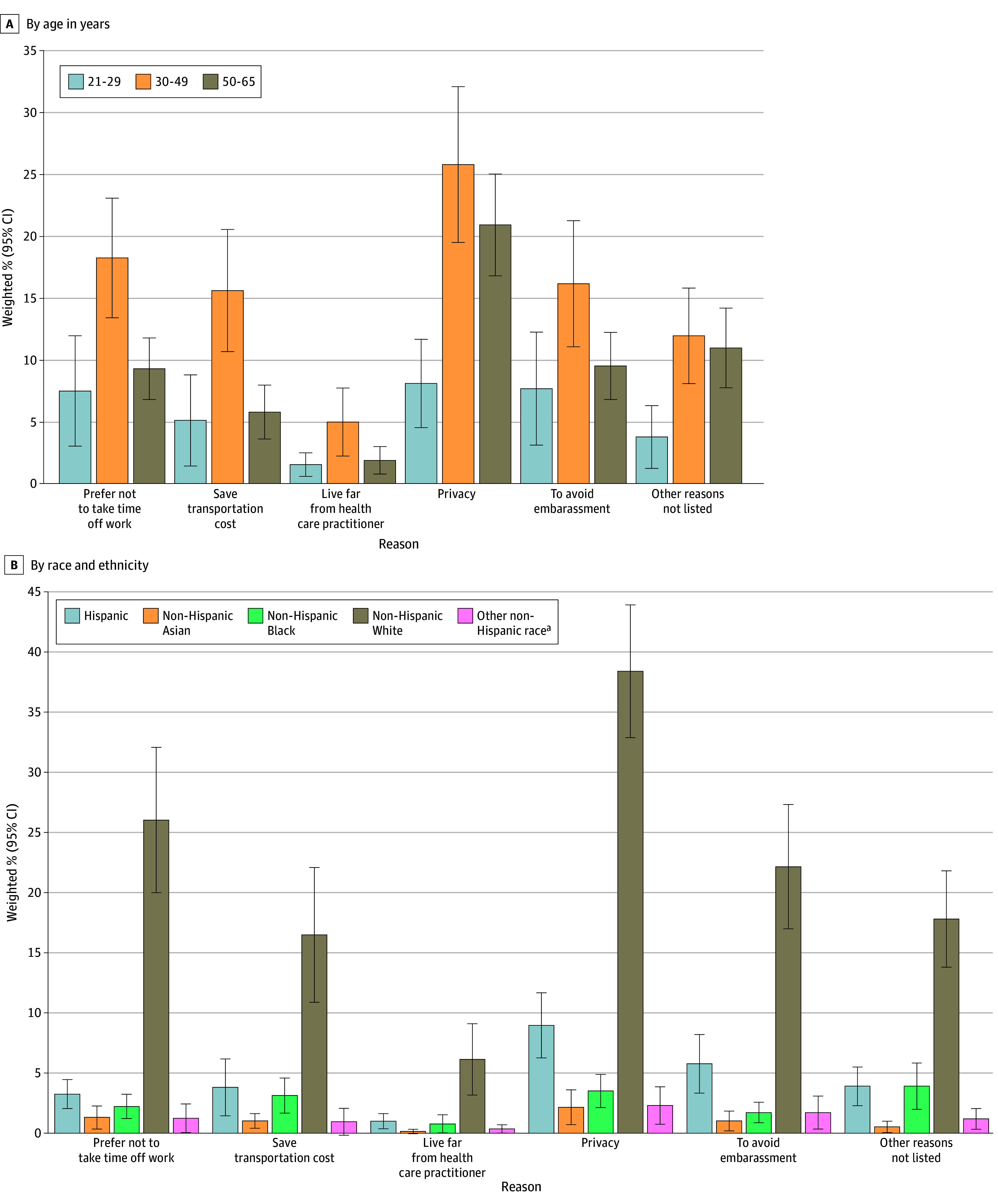
Bar Graphs of Reasons for Considering Home-Based Self-Sampling for Cervical Cancer Screening Among Eligible US Women in the 2024 Health Interview National Trends Survey, by Age and Race and Ethnicity Error bars represent 95% CIs. Numerators were the number of participants who selected each reason for considering at-home self-sampling and denominators were the total number of participants who responded to the question and preferred at-home self-sampling or did not know which option to choose. Because respondents could select more than 1 response, percentages sum to more than 100. ^a^Included individuals of the following minority race groups who reported their ethnicity as non-Hispanic: American Indian or Alaska Native; Guamanian or Chamorro; or Native Hawaiian, Samoan, or Other Pacific Islander.

Among women who preferred at-home self-sampling or who were uncertain about their choice (n = 898 [39.2%; 95% CI, 35.6%-42.8%]), the most commonly reported reasons for considering at-home self-sampling were privacy (54.9%; 95% CI, 49.8%-60.0%), time constraints (35.1%; 95% CI, 29.0%-41.2%), avoiding embarrassment (33.4%; 95% CI, 28.0%-38.8%), and transportation cost (26.5%; 95% CI, 20.1%-32.9%) (Figure 1). The reasons for considering at-home self-sampling varied according to certain characteristics, including age and race and ethnicity (Figure 2).

## Discussion

In this population-based cross-sectional study of women eligible for cervical cancer screening, a lower proportion of individuals reported preference for home-based self-sampling compared with those who reported preference for clinic-based testing. The most commonly reported reasons for considering at-home self-sampling were privacy, time constraints, and fear of embarrassment. To our knowledge, this study is among the first to nationally examine perspectives about and preferences for an innovative screening strategy based on at-home self-sampling compared with clinician-collected sampling (the current standard of care) among US women eligible for cervical cancer screening.

Since women who reported preference for at-home self-sampling were more likely to be overdue for cervical cancer screening,^37,38^ offering this alternative screening option to the general population could help close the screening gap in women who are hesitant to receive conventional (clinic-based) cervical cancer screening due to the various access barriers to health care services, thereby reducing disparities in cervical cancer screening uptake in the US.

Concerning women’s preferences, our findings echo other studies conducted in specific populations enrolled in clinical trials.^21,22,23,24,39^ Among underscreened women participating in a study to assess the cost-effectiveness and acceptability of mailed self-sampling kits in a safety net health system in the US,^21^ most participants (84.8%) reported a positive experience with self-sampling. In that study, self-sampling helped overcome many barriers to clinic-based testing and was perceived by most women as more convenient (89.0%) or less embarrassing (99.4%) than clinic-based testing.^21^ In another US-based pilot trial aimed at evaluating the effectiveness of a culturally adapted educational intervention to promote self-sampling use among racial and ethnic minority groups and how this innovative screening approach can affect cervical cancer screening uptake in underscreened populations, a majority of women (75%) who had ever had clinic-based testing found HPV self-sampling more convenient, and 92% preferred at-home self-sampling.^23^ In a third US study—a clinical trial to validate a new self-collection tool for at-home use in a diverse population of screening-eligible individuals^24^—this alternative screening modality was reported to be acceptable to the study population, who found that it would help overcome several of the barriers they encountered in accessing conventional cervical cancer screening. One-third of participants (31.9%) reported delaying or avoiding screening, while 93% indicated that they would prefer self-sampling.^24^ In that study, racial and ethnic minority individuals and socioeconomically disadvantaged groups, who are known to have lower participation in guideline-based screening,^32^ were more likely to accept at-home self-sampling. In another study comparing the performance of a self-sampling device with clinician-collected samples among women referred for colposcopy at a tertiary-level health care facility in Canada, a considerable proportion of enrolled women (56.6%) preferred self-sampling over clinician-collected sampling, especially when instructed on how to collect the sample properly.^39^ Of note, only 37.8% of women who had performed both self-sampling and clinic-based testing as part of that trial reported that they preferred clinician-collected sampling, while 5% had no preference.^39^

At a time when the USPSTF and the ACS, along with other scholarly organizations, are revising their guidelines for cervical cancer screening to determine how to incorporate at-home HPV self-sampling as a recommended screening modality in the US, our findings provide insights from women’s perspectives that could inform new cervical cancer screening guidelines and guide implementation policies nationwide. Following the surge of the COVID-19 pandemic, many countries have updated their screening recommendations to include HPV self-sampling as an alternative to clinic-based testing.^40^ With the recent approval of an HPV self-sampling device for at-home use by the FDA, and considering that the use of home-based self-sampling kits has been shown to be a cost-effective intervention that can increase screening uptake among underscreened populations,^22,23,41,42^ the integration of this innovative approach into US screening guidelines and its adoption by cervical cancer screening programs is imperative. As reported in the literature, an essential factor influencing the adoption and implementation of an evidence-based health care intervention is its perception and acceptability by the target population.^43^

Our findings that women of racial or ethnic minority groups were more likely to prefer clinic-based testing over home-based self-sampling and less likely to be uncertain about their choice corroborate previous reports^44,45,46,47^ and suggest a lower level of self-confidence in performing self-sampling in the absence of a health care practitioner or outside the medical setting among these individuals, who are paradoxically at higher risk of cervical cancer.^48^ Therefore, targeted and culturally adapted interventions aimed at educating these women on the safety and effectiveness of at-home self-sampling are needed to reduce cervical cancer disparities and increase screening coverage in hard-to-reach populations. A tailored educational intervention adapted to low-income non-Hispanic and Black women that has recently been piloted in the state of Texas has resulted in increased cervical cancer screening uptake in this population.^23^ Another pilot study conducted in an integrated health care delivery system in the state of Washington reported improvements in cervical cancer screening participation among women who received HPV self-sampling kits mailed to their home compared with those assigned to schedule clinic-based testing.^22,42^ Such strategies should be further evaluated across different states and among diverse populations, with a particular focus on medically underserved and hard-to-reach communities, to determine the suitable implementation approach for these populations.

A key finding of this study was that women who had experienced discrimination when seeking care and those who had little to no trust in the health care system had higher odds of preferring at-home self-sampling and of being uncertain about their choice, respectively. In the US, perceived discrimination in medical settings and lack of trust in the health care system are associated with lower uptake and adherence to cervical cancer screening, especially among racial and ethnic minority groups.^49,50^ According to the World Health Organization’s Consolidated Guideline on Self-Care Interventions for Health, self-care has the readily apparent benefits of privacy, confidentiality, speed, convenience, and access.^51^ It is “people-centered” and enables active participation in one’s own health. It is also a health system approach that can reduce the burden on strained systems with shortages in medical personnel or other barriers to health care access. Therefore, our findings underscore the value of strategies based on home-based self-sampling in reducing access barriers and improving adherence to cervical cancer screening among women who are reluctant to go for screening because of perceived discrimination and mistrust of the health care system. The fact that age, urbanicity of place of residence, and educational level were not associated with preference for home-based self-sampling suggests that women’s perceptions about this innovative screening strategy encompass multiple generations and educational backgrounds.

To reverse the declining trends observed in cervical cancer screening adherence in the past 2 decades in the US, breakthrough approaches, such as home-based self-sampling, that can potentially reduce health disparities by increasing screening participation among hard-to-reach and underscreened populations should be adopted and implemented. Future studies conducted in routine clinical settings are needed to understand the mechanisms that underpin the observed association between individual-, community-, and system-level factors and women’s preference for at-home self-sampling.

Although there were higher odds of preferring at-home self-sampling among certain sociodemographic minority populations, we did not find statistically significant associations with characteristics such as lack of health insurance coverage, rural dwelling, and gender identity that have consistently been associated with lower cervical cancer screening rates.^31,32,52^ The lack of statistical significance for these variables could be explained by the confounding effect of other covariates (such as perceived discrimination in the medical setting, educational level, household income, and race and ethnicity) on one hand and on the other hand, the uneven distribution in our study sample of respondents pertaining to some categories of these variables, which may have contributed to lessening the statistical power to detect an association with preference for at-home self-sampling. For example, most participants were health-insured (91.9%), heterosexual (87.2%), or residing in urban areas (85.6%), suggesting a possible underrepresentation of gender minority individuals, uninsured individuals, and rural dwellers in the study sample.

### Limitations

Our study has limitations. First, it was limited by the cross-sectional design of the survey, which precluded us from establishing a causal relationship between women’s preferences for home-based self-sampling and associated factors. Second, it is likely that some respondents to the HINTS 7 were unfamiliar with this new screening modality, as at-home self-sampling was neither endorsed by US screening guidelines nor recommended by health care practitioners at the time of the survey. Accordingly, our findings helped characterize the population of individuals who may be early adopters of this alternative screening modality and who should be targeted by developers of sampling devices (eg, vaginal brush or swab) that can be used to implement at-home self-sampling to further refine their products and improve their safety and acceptability as perceived by the population. Additionally, self-sampling is a technique that has been widely used for the testing of sexually transmitted infections in the US over the past decades, and its use has increased since the onset of the COVID-19 pandemic.^53^ Therefore, many study participants who were eligible for cervical cancer screening may have experienced or been exposed to self-testing for other purposes. Third, the survey used in this study was administered a few months before the FDA approved the first HPV test for at-home self-sampling, which may have contributed to the relatively high proportion (about one-fifth) of respondents who indicated uncertainty about their choice. Fourth, because data on prior screening history and experiences were not collected as part of HINTS 7, this potential confounder could not be included in our analysis. Although the applicability of our findings to women who are up-to-date on cervical cancer screening could not be ascertained, prior studies have reported that women who experience discrimination, whether racially based, gender based, or other forms, are significantly more likely to be underscreened or overdue for cervical cancer screening.^32,54,55^ Therefore, women who reported preference for at-home self-sampling in this study’s population were less likely to be up-to-date on cervical cancer screening.

Despite these limitations, the originality of this study lies in its use of a nationally representative sample of women eligible for cervical cancer screening to assess their perspectives about at-home self-sampling. In 2024, the HINTS introduced for the first time a set of questions about self-sampling, which served as the basis for this population-based study. By examining sociodemographic and health-behavioral factors influencing individuals’ preferences for self-sampling, our study provides valuable insights into the adoption of this new technology, which has the potential to improve access to cervical cancer screening among underserved communities.

## Conclusions

In this cross-sectional study of individuals eligible for cervical cancer screening in the US, about one-fifth of participants reported preference for home-based self-sampling over clinic-based testing, and a similar proportion reported not knowing which sampling option to choose, with marginalized groups, individuals who do not trust the health care system, and sociodemographically disadvantaged populations being more likely to consider at-home self-sampling or to be unsure about their choice. To address cervical cancer inequities and increase screening uptake among underscreened populations, at-home self-sampling should be incorporated into US screening guidelines as an alternative to clinic-based testing, endorsed by the medical community and scholarly organizations, and implemented at a larger scale. To foster the adoption of home-based self-sampling and provide more options to women for whom cervical cancer screening is recommended, women’s education and empowerment should be enhanced, and tailored interventions and informational campaigns aimed at promoting this novel screening modality are needed, particularly focusing on high-risk groups such as non-Hispanic Black individuals and women who experience prejudice or discrimination when seeking medical care.

## Notes

Supplement.
Data Sharing Statement

## References

[1] Noone AM, Howlader N, Krapcho M, , eds. SEER cancer statistics review, 1975-2015. National Cancer Institute. April 2018. Accessed May 13, 2025. https://seer.cancer.gov/csr/1975_2015/

[2] US National Cancer Institute. Cancer trends progress report: cervical cancer screening. Accessed May 15, 2025. https://progressreport.cancer.gov/detection/cervical_cancer

[3] American Cancer Society. Cancer facts and figures, 2024. 2024. Accessed May 15, 2025. https://www.cancer.org/content/dam/cancer-org/research/cancer-facts-and-statistics/annual-cancer-facts-and-figures/2024/2024-cancer-facts-and-figures-acs.pdf

[4] Surveillance, Epidemiology, and End Results Program. Cancer stat facts: cervical cancer. US National Cancer Institute. Accessed May 15, 2025. https://seer.cancer.gov/statfacts/html/cervix.html

[5] Beavis AL, Gravitt PE, Rositch AF. Hysterectomy-corrected cervical cancer mortality rates reveal a larger racial disparity in the United States. Cancer. 2017;123(6):1044-1050. doi:10.1002/cncr.30507 28112816

[6] Leyden WA, Manos MM, Geiger AM, . Cervical cancer in women with comprehensive health care access: attributable factors in the screening process. J Natl Cancer Inst. 2005;97(9):675-683. doi:10.1093/jnci/dji115 15870438

[7] International Agency for Research on Cancer. Cervix Cancer Screening. IARC Press; 2005. IARC Handbooks of Cancer Prevention; vol 10.

[8] Ogilvie GS, van Niekerk D, Krajden M, . Effect of screening with primary cervical HPV testing vs cytology testing on high-grade cervical intraepithelial neoplasia at 48 months: the HPV FOCAL randomized clinical trial. JAMA. 2018;320(1):43-52. doi:10.1001/jama.2018.7464 29971397 PMC6583046

[9] Ronco G, Giorgi-Rossi P, Carozzi F, ; New Technologies for Cervical Cancer screening (NTCC) Working Group. Efficacy of human papillomavirus testing for the detection of invasive cervical cancers and cervical intraepithelial neoplasia: a randomised controlled trial. Lancet Oncol. 2010;11(3):249-257. doi:10.1016/S1470-2045(09)70360-2 20089449

[10] Curry SJ, Krist AH, Owens DK, ; US Preventive Services Task Force. Screening for cervical cancer: US Preventive Services Task Force recommendation statement. JAMA. 2018;320(7):674-686. doi:10.1001/jama.2018.10897 30140884

[11] Chor J, Davis AM, Rusiecki JM. Cervical cancer screening guideline for individuals at average risk. JAMA. 2021;326(21):2193-2194. doi:10.1001/jama.2021.13448 34766970

[12] Arbyn M, Smith SB, Temin S, Sultana F, Castle P; Collaboration on Self-Sampling and HPV Testing. Detecting cervical precancer and reaching underscreened women by using HPV testing on self samples: updated meta-analyses. BMJ. 2018;363:k4823. doi:10.1136/bmj.k4823 30518635 PMC6278587

[13] Polman NJ, Ebisch RMF, Heideman DAM, . Performance of human papillomavirus testing on self-collected versus clinician-collected samples for the detection of cervical intraepithelial neoplasia of grade 2 or worse: a randomised, paired screen-positive, non-inferiority trial. Lancet Oncol. 2019;20(2):229-238. doi:10.1016/S1470-2045(18)30763-0 30658933

[14] Limmer K, LoBiondo-Wood G, Dains J. Predictors of cervical cancer screening adherence in the United States: a systematic review. J Adv Pract Oncol. 2014;5(1):31-41. 25032031 PMC4093462

[15] Crawford A, Benard V, King J, Thomas CC. Understanding barriers to cervical cancer screening in women with access to care, behavioral risk factor surveillance system, 2014. Prev Chronic Dis. 2016;13:E154. doi:10.5888/pcd13.160225 27831682 PMC5109933

[16] Benard VB, Thomas CC, King J, Massetti GM, Doria-Rose VP, Saraiya M; Centers for Disease Control and Prevention (CDC). Vital signs: cervical cancer incidence, mortality, and screening—United States, 2007-2012. MMWR Morb Mortal Wkly Rep. 2014;63(44):1004-1009. 25375072 PMC5779486

[17] Verdoodt F, Jentschke M, Hillemanns P, Racey CS, Snijders PJ, Arbyn M. Reaching women who do not participate in the regular cervical cancer screening programme by offering self-sampling kits: a systematic review and meta-analysis of randomised trials. Eur J Cancer. 2015;51(16):2375-2385. doi:10.1016/j.ejca.2015.07.006 26296294

[18] Winer R, Tiro JA, Miglioretti DL, . Corrigendum to “rationale and design of the HOME trial: a pragmatic randomized controlled trial of home-based human papillomavirus (HPV) self-sampling for increasing cervical cancer screening uptake and effectiveness in a US healthcare system” [Contemp. Clin. Trials 64 (2018) 77-87]. Contemp Clin Trials. 2019;84:105811. doi:10.1016/j.cct.2019.07.003 31307906

[19] Pretsch PK, Spees LP, Brewer NT, . Effect of HPV self-collection kits on cervical cancer screening uptake among under-screened women from low-income US backgrounds (MBMT-3): a phase 3, open-label, randomised controlled trial. Lancet Public Health. 2023;8(6):e411-e421. doi:10.1016/S2468-2667(23)00076-2 37182529 PMC10283467

[20] FDA approves Teal Health’s Teal Wand—the first and only at-home self-collection device for cervical cancer screening, introducing a comfortable alternative to in-person screening. News release. Teal Health; May 9, 2025. Accessed May 15, 2025. https://www.getteal.com/news/fda-approves-teal-healths-teal-wand-tm---the-first-and-only-at-home-self-collection-device-for-cervical-cancer-screening-introducing-a-comfortable-alternative-to-in-person-screening

[21] Parker SL, Amboree TL, Bulsara S, . Self-sampling for human papillomavirus testing: acceptability in a US safety net health system. Am J Prev Med. 2024;66(3):540-547. doi:10.1016/j.amepre.2023.10.020 37935320 PMC12177980

[22] Winer RL, Lin J, Anderson ML, . Strategies to increase cervical cancer screening with mailed human papillomavirus self-sampling kits: a randomized clinical trial. JAMA. 2023;330(20):1971-1981. doi:10.1001/jama.2023.21471 38015219 PMC10685881

[23] Shastri SS, McNeill LH, Shete S. Culturally competent education and human papillomavirus self-sampling achieves Healthy People 2030 cervical screening target among low-income non-Hispanic Black and Hispanic women. JCO Glob Oncol. 2024;10:e2400005. doi:10.1200/GO.24.00005 38723214 PMC11929156

[24] Fitzpatrick MB, Behrens CM, Hibler K, . Clinical validation of a vaginal cervical cancer screening self-collection method for at-home use: a nonrandomized clinical trial. JAMA Netw Open. 2025;8(5):e2511081. doi:10.1001/jamanetworkopen.2025.11081 40388167 PMC12090030

[25] Health Information National Trends Survey 7 (HINTS 7) methodology report. Accessed November 30, 2025. https://hints.cancer.gov/docs/methodologyreports/HINTS_7_MethodologyReport.pdf

[26] Office for Human Research Protections. Regulations, policy & guidance. US Department of Health and Human Services 2022. Accessed March 3, 2024. https://www.hhs.gov/ohrp/regulations-and-policy/index.html

[27] EQUATOR (Enhancing the Quality and Transparency of Health Research) Network. Reporting guidelines for main study types. Accessed May 12, 2025. https://www.equator-network.org/

[28] McLeroy KR, Bibeau D, Steckler A, Glanz K. An ecological perspective on health promotion programs. Health Educ Q. 1988;15(4):351-377. doi:10.1177/109019818801500401 3068205

[29] Herman JL, Flores AR. How many adults and youth identify as transgender in the United States? UCLA School of Law Williams Institute. August 2025. Accessed December 10, 2025. https://williamsinstitute.law.ucla.edu/publications/trans-adults-united-states/

[30] Gorina Y, Elgaddal N, Weeks JD. Hysterectomy among women age 18 and older: United States, 2021. NCHS Data Brief no 494. National Center for Health Statistics. February 28, 2024. 38421296

[31] Borders TF, Thaxton Wiggins A. Cervical cancer screening rates among rural and urban females, from 2019 to 2022. JAMA Netw Open. 2024;7(6):e2417094. doi:10.1001/jamanetworkopen.2024.17094 38874926 PMC11179126

[32] Fokom Domgue J, Chandra M, Yu R, Shete S. Adherence to cervical cancer screening among sexual and racial/ethnic minorities during the COVID-19 pandemic in the United States. Am J Obstet Gynecol. 2025;232(5):e174-e179. doi:10.1016/j.ajog.2025.02.008 39923875

[33] Alba C, Zheng Z, Wadhera RK. Changes in health care access and preventive health screenings by race and ethnicity. JAMA Health Forum. 2024;5(2):e235058. doi:10.1001/jamahealthforum.2023.5058 38306093 PMC10837752

[34] Suk R, Hong YR, Rajan SS, Xie Z, Zhu Y, Spencer JC. Assessment of US Preventive Services Task Force guideline–concordant cervical cancer screening rates and reasons for underscreening by age, race and ethnicity, sexual orientation, rurality, and insurance, 2005 to 2019. JAMA Netw Open. 2022;5(1):e2143582. doi:10.1001/jamanetworkopen.2021.43582 35040970 PMC8767443

[35] Spencer JC, Kim JJ, Tiro JA, . Racial and ethnic disparities in cervical cancer screening from three US healthcare settings. Am J Prev Med. 2023;65(4):667-677. doi:10.1016/j.amepre.2023.04.016 37146839 PMC11135625

[36] Du R, Choi YJ, Lee JH, Songthip O, Hu Z. A weighted jackknife approach utilizing linear model based-estimators for clustered data. Commun Stat Simul Comput. 2024;53(2):1048-1067. doi:10.1080/03610918.2022.2039396 38523866 PMC10959512

[37] Qin J, Martinez G, Holt HK, . Preferences among US women for cervical cancer screening with self-collected specimens for human papillomavirus testing. Obstet Gynecol. 2025. doi:10.1097/AOG.0000000000006147 41380156 PMC12797866

[38] Kilfoyle KA, Des Marais AC, Ngo MA, . Preference for human papillomavirus self-collection and Papanicolaou: survey of underscreened women in North Carolina. J Low Genit Tract Dis. 2018;22(4):302-310. doi:10.1097/LGT.0000000000000430 30179994 PMC6174678

[39] El-Zein M, Bouten S, Louvanto K, ; CASSIS Study Group. Validation of a new HPV self-sampling device for cervical cancer screening: the Cervical and Self-Sample In Screening (CASSIS) study. Gynecol Oncol. 2018;149(3):491-497. doi:10.1016/j.ygyno.2018.04.004 29678360

[40] Serrano B, Ibáñez R, Robles C, Peremiquel-Trillas P, de Sanjosé S, Bruni L. Worldwide use of HPV self-sampling for cervical cancer screening. Prev Med. 2022;154:106900. doi:10.1016/j.ypmed.2021.106900 34861338

[41] Winer RL, Lin J, Tiro JA, . Effect of mailed human papillomavirus test kits vs usual care reminders on cervical cancer screening uptake, precancer detection, and treatment: a randomized clinical trial. JAMA Netw Open. 2019;2(11):e1914729. doi:10.1001/jamanetworkopen.2019.14729 31693128 PMC6865279

[42] Winer RL, Lin J, Tiro JA, . Effect of patient characteristics on uptake of screening using a mailed human papillomavirus self-sampling kit: a secondary analysis of a randomized clinical trial. JAMA Netw Open. 2022;5(11):e2244343. doi:10.1001/jamanetworkopen.2022.44343 36449291 PMC9713609

[43] Klaic M, Kapp S, Hudson P, . Implementability of healthcare interventions: an overview of reviews and development of a conceptual framework. Implement Sci. 2022;17(1):10. doi:10.1186/s13012-021-01171-7 35086538 PMC8793098

[44] Mkuu RS, Staras SA, Chakrabarti C, . Acceptability of HPV self-collection: a qualitative study of Black women living with type II diabetes and social vulnerability. J Clin Transl Endocrinol. 2024;35:100331. doi:10.1016/j.jcte.2024.100331 38444842 PMC10912756

[45] Anhang R, Nelson JA, Telerant R, Chiasson MA, Wright TC Jr. Acceptability of self-collection of specimens for HPV DNA testing in an urban population. J Womens Health (Larchmt). 2005;14(8):721-728. doi:10.1089/jwh.2005.14.721 16232104

[46] Kahn JA, Bernstein DI, Rosenthal SL, . Acceptability of human papillomavirus self testing in female adolescents. Sex Transm Infect. 2005;81(5):408-414. doi:10.1136/sti.2004.012047 16199741 PMC1745047

[47] Guan Y, Castle PE, Wang S, . A cross-sectional study on the acceptability of self-collection for HPV testing among women in rural China. Sex Transm Infect. 2012;88(7):490-494. doi:10.1136/sextrans-2012-050477 22645391

[48] Surveillance, Epidemiology, and End Results Program. SEER Incidence Data, 1975-2022. National Cancer Institute. Accessed May 15, 2025. https://seer.cancer.gov/data/

[49] Washington A, Randall J. “We’re not taken seriously”: describing the experiences of perceived discrimination in medical settings for Black women. J Racial Ethn Health Disparities. 2023;10(2):883-891. doi:10.1007/s40615-022-01276-9 35239178 PMC8893054

[50] Jacobs EA, Rathouz PJ, Karavolos K, . Perceived discrimination is associated with reduced breast and cervical cancer screening: the Study of Women’s Health Across the Nation (SWAN). J Womens Health (Larchmt). 2014;23(2):138-145. doi:10.1089/jwh.2013.4328 24261647 PMC3922246

[51] World Health Organization. *WHO Guideline on Self-Care Interventions for Health and Well-Being, 2022 Revision*. 2022. Accessed July 12, 2025. https://www.ncbi.nlm.nih.gov/books/NBK582359/35914064

[52] Baumann K, Matzke H, Peterson CE, . Sexual orientation and cervical cancer screening among cisgender women. JAMA Netw Open. 2024;7(5):e248886. doi:10.1001/jamanetworkopen.2024.8886 38709536 PMC11074807

[53] Spence T, Howarth A, Reid D, . How does online postal self-sampling (OPSS) shape access to testing for sexually transmitted infections (STIs)? a qualitative study of service users. BMC Public Health. 2024;24(1):2339. doi:10.1186/s12889-024-19741-x 39198751 PMC11360737

[54] Hernandez AE, Borowsky PA, Lubarsky M, . Associations between perceived discrimination, screening mammography, and breast cancer stage at diagnosis: a prospective cohort analysis. Ann Surg Oncol. 2024;31(12):8012-8020. doi:10.1245/s10434-024-15757-0 39060693 PMC11467043

[55] Moscoso-Porras MG, Alvarado GF. Association between perceived discrimination and healthcare-seeking behavior in people with a disability. Disabil Health J. 2018;11(1):93-98. doi:10.1016/j.dhjo.2017.04.002 28420592

