# Supplementary material for: Women’s Preferences for Home-Based Self-Sampling or Clinic-Based Testing for Cervical Cancer Screening
Source: JAMA Netw Open. 2026 Feb 6;9(2):e2558841. doi: 10.1001/jamanetworkopen.2025.58841 (PMC12881987; doi:10.1001/jamanetworkopen.2025.58841)
Supplement: Supplement. — Data Sharing Statement [file jamanetwopen-e2558841-s001.pdf]

Data Sharing Statement

Fokom Domgue. Women’s Preferences for Home-Based Self-Sampling or Clinic-Based Testing for Cervical Cancer Screening. *JAMA Netw Open*. Published February 06, 2026. doi:10.1001/jamanetworkopen.2025.58841

Data
Data available: No
